# Development of a Novel Methodology for Remaining Useful Life Prediction of Industrial Slurry Pumps in the Absence of Run to Failure Data

**DOI:** 10.3390/s21248420

**Published:** 2021-12-16

**Authors:** Muhammad Mohsin Khan, Peter W. Tse, Amy J. C. Trappey

**Affiliations:** 1 Department of Advanced Design and Systems Engineering, City University of Hong Kong, Hong Kong, China; muhakhan2-c@my.cityu.edu.hk; 2 Department of Industrial Engineering and Engineering Management, National Tsing Hua University, Hsinchu 300, Taiwan; trappey@ie.nthu.edu.tw

**Keywords:** slurry pumps, remaining useful life prediction, LSTM-BiLSTM model

## Abstract

Smart remaining useful life (RUL) prognosis methods for condition-based maintenance (CBM) of engineering equipment are getting high popularity nowadays. Current RUL prediction models in the literature are developed with an ideal database, i.e., a combination of a huge “run to failure” and “run to prior failure” data. However, in real-world, run to failure data for rotary machines is difficult to exist since periodic maintenance is continuously practiced to the running machines in industry, to save any production downtime. In such a situation, the maintenance staff only have run to prior failure data of an in operation machine for implementing CBM. In this study, a unique strategy for the RUL prediction of two identical and in-process slurry pumps, having only real-time run to prior failure data, is proposed. The obtained vibration signals from slurry pumps were utilized for generating degradation trends while a hybrid nonlinear autoregressive (NAR)-LSTM-BiLSTM model was developed for RUL prediction. The core of the developed strategy was the usage of the NAR prediction results as the “path to be followed” for the designed LSTM-BiLSTM model. The proposed methodology was also applied on publically available NASA’s C-MAPSS dataset for validating its applicability, and in return, satisfactory results were achieved.

## 1. Introduction

Smart methods of remaining useful life (RUL) prediction for condition-based maintenance (CBM) of engineering equipment are getting a high demand in the modern industrial era. As per the current literature, it was observed that the majority of the RUL-related works have been performed by utilizing the publically available “ideal” datasets. These datasets, for instance, Commercial Modular Aero-Propulsion System Simulation (C-MAPSS) dataset or PRONOSTIA bearing dataset, etc. These datasets consist of huge training (run to failure) data, and testing (run to prior failure) data. Typically, researchers utilized the available “run to failure” data for the learning stage of their developed model, while the “run to prior failure” data for checking the accuracy of their developed models. However, in real-world run to failure data for equipment is typically not available in the industry. Since time-based maintenance (TBM) is performed periodically on a machine with the aim to save it from failure or production downtime, in such a situation, the maintenance officer needs to only rely on the available run to prior failure data of an in operation machine in order to implement CBM. In this study, a unique strategy for the RUL prediction of two identical and in operation slurry pumps, having only run to prior failure data in their real-time, has been established.

Slurry pumps are widely used in mining, waste treatment, and oil sand industries for the purpose of dragging and propelling the mixtures of abrasive liquids and solids. Intensive wear on pump components is typically induced by the “pumped” abrasive and erosive mixtures. Components that are most prominently affected and get degraded are the impellers of the slurry pumps due to their direct contact with slurry mixtures [1]. Once slurry pump impellers fail during the operation, it creates major operational breakdown and significant economic loss to the industry. An unexpected shut-down of a slurry pump can cost $1.3 million per day in lost oil production [2]. In industrial practices, it is assumed that if an operational slurry pump has been found to be running anomalously, as indicated by its sound or vibration data, etc., then the severity of damage caused to the pump should be diagnosed. Therefore, the RUL of the deteriorating slurry pump should be estimated before its sudden or fatal breakdown. Keeping in view the situation, this research aimed to (i) analyze the obtained real-time vibration datasets from the slurry pumps, which were obtained by the four mounted accelerometers, in the format of time series data, in the year 2014 and 2017 (ii) generate the health indicators/performance degradation trends of the slurry pumps using their vibration data, and (iii) to develop a robust prediction model that can predict online, not only the overall RUL but also the short-term RUL of the degrading slurry pumps with acceptable accuracy.

While going through the literature, it was found that only four researchers, i.e., [3,4,5,6], have estimated the RUL of slurry pumps. However, their work was deficient in terms of applicability since all of them used the curve fitting technique for RUL prediction. It was noticed that their degradation trends were progressing towards the threshold line in a very smooth way. Technically it means that tiny stones presented in the “pumped slurry” that were actually hitting the impellers were comparatively of uniform size. However, it is not the case every time. Instead, in most of the scenarios, the slurry particles are of varying size, which hit the slurry pumps impellers with a very high speed in an irregular period of time. For such a situation, the degradation trend generated for that particular slurry pump will have many deep crest and trough-like structures. In that case, RUL estimated via the curve fitting technique is not appropriate. Hence, a new robust approach that can predict the online RUL of slurry pumps with its applicability even for the gradually increasing degradation trends, with many deep crest and trough-like structures, is presented in this research. The new approach is based on nonlinear autoregressive (NAR) networks and deep learning-based LSTM and BiLSTM networks due to their excellent abilities in grabbing the characteristics of the collected datasets. In order to predict the RUL of the slurry pumps, the first few prediction points were delivered by the NAR model. Then those prediction points were followed by the designed LSTM-BiLSTM model for predicting the short-term and overall RULs. The detail of the novel strategy has been discussed in Section 2.3 of this paper. In order to check the validity of the proposed method, it has also been applied on (i) other channels of the slurry pumps, and (ii) two individual turbofan jet engines of publically available C-MAPSS dataset by only utilizing their given run to prior failure data, as per the concept explained above. RUL prediction results obtained by the developed model also showed their dominance when they were compared with another machine learning technique of online RUL prediction.

Background knowledge regarding NAR, LSTM, and BiLSTM models and their current application for solving the engineering problems are precisely discussed in Sections S1 and S2.

## 2. Methodology Adopted for Predicting the RUL of Slurry Pumps

### 2.1. Data Collection and the Determination of Valid Data Sets

**Figure 1:**
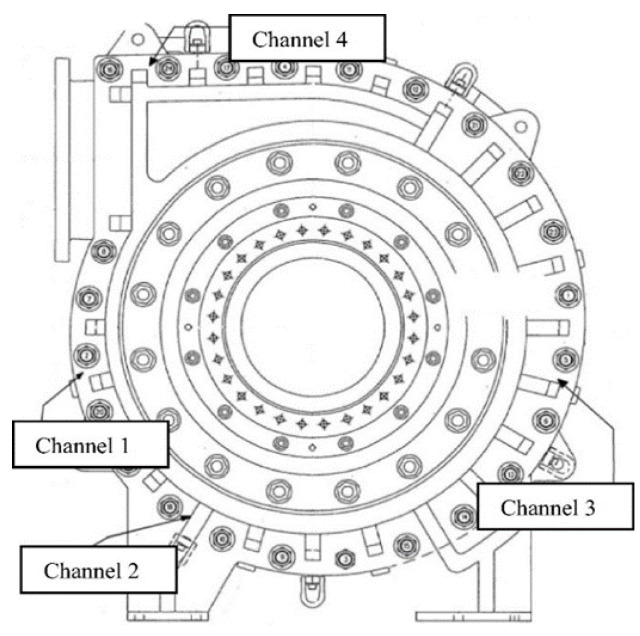
The measurement locations of the four accelerometers mounted on a slurry pump casing (the locations of the four accelerometers named as channel 1, 2, 3, and 4) [5].

Since, for inquiring the health condition of a pump, analyses of its vibration measurements is a simple and direct way [7]. Therefore, to determine the level of wear/degradation to slurry pumps, Peter Tse and his research team conducted a series of experiments on on-site operating slurry pumps [8] and decided on the most appropriate locations for mounting sensors on the considered slurry pump. They mounted four PCB 352A60 (Kistler Instrument Corp., Hudson Drive, 48377 Novi, MI, USA)accelerometers (sensors) on four specified locations of a slurry pump for capturing the vibration signals, as shown in Figure 1.

This procedure was performed twice. First, in the year 2014, and then in 2017 on an identical slurry pump. A heavy motor, having an initial rotation frequency of 26 Hz was driving the oil sand pump, which got reduced to a rotation frequency of 6.63 Hz after a connection with a gearbox. Since there were four blades in the impellers, therefore the vane-passing frequency became 26.48 Hz., while the calculated first and second harmonic frequency of the tooth meshing frequency was found to be 364 Hz and 728 Hz, respectively [5]. A sensor signal analyzing and data acquisition software named as Smart Asset Management System (SAMS), i.e., developed by National Instrument’s (NI’s) Labview tool, was utilized for collecting the data. The frequency range of sensors was between 5 Hz to 60 kHz, while the sensitiveness of channel 4, channel 3, channel 2, and channel 1, was 10.10, 10.11, 10.17, and 10.13, respectively. Vibration generated at the output of the slurry pump was monitored by channel 4. Channel 2 and channel 3 monitored the vibrations which were produced by the impellers passing vanes while the vibration generated at the input or suction of the slurry pump was monitored by channel 1. Each slurry pump was overhauled with the replacement of its internal components (except impellers) that had been worn out substantially during the data-taking process. The pumps remain in operation for around three months in ON/OFF condition until their scheduled time for maintenance, i.e., TBM, would not reach. The sampling frequency rate of vibration signals was found to be 51.2 kHz for the first slurry pump (the year 2014) and 60 kHz for the second slurry pump (the year 2017). Since it was obvious that data accumulated from sensors always have some noise, therefore, vibration signals which were getting out of a predefined threshold were cleaned manually.

### 2.2. Development of Vibration-Based Degradation Trends

**Figure 2:**
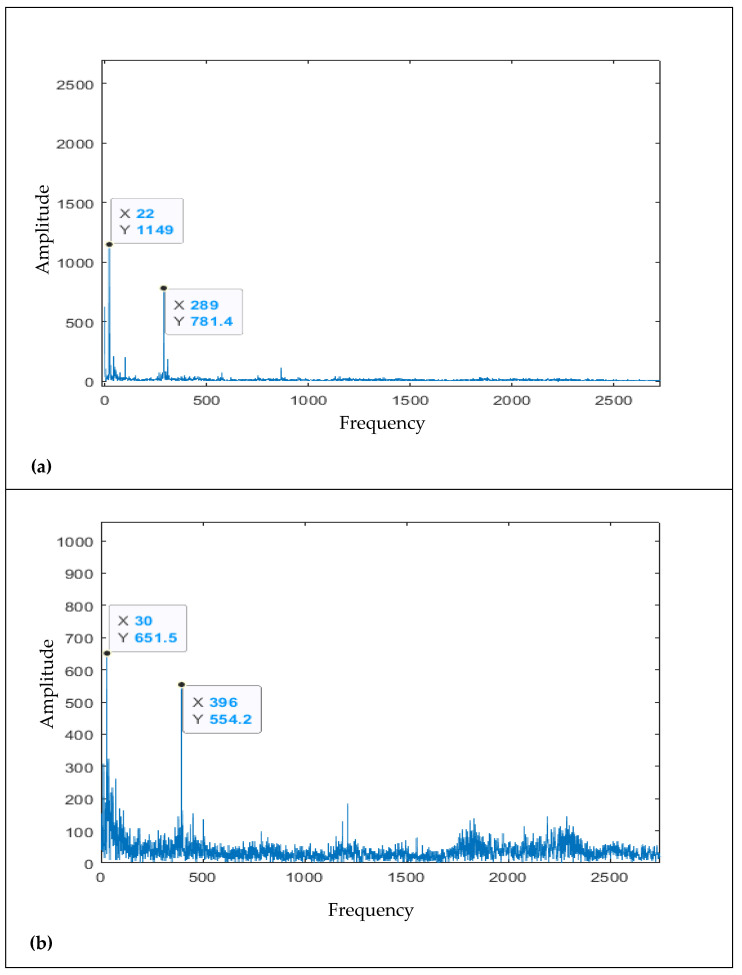

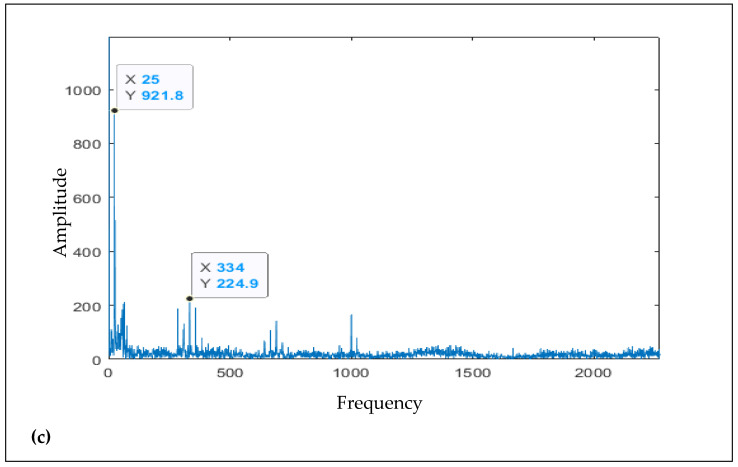
The frequency spectra of the vibrations collected for time measurement (**a**) 58, (**b**) 650, (**c**) 892 for channel 3 of the 2014 datasets.

As mentioned earlier, the vanes of impellers are typically the component of slurry pumps that wear out mainly due to their direct contact with sand and solid particles of slurry. Therefore, the conducted research has specifically focused on the impellers of slurry pumps. The raw vibration data obtained from channel 3, from both dataset 2014 and dataset 2017, were utilized for conducting this research. Figure 2 shows frequency spectra of the vibrations collected for some time measurements for channel 3 of 2014 datasets.

**Figure 3:**
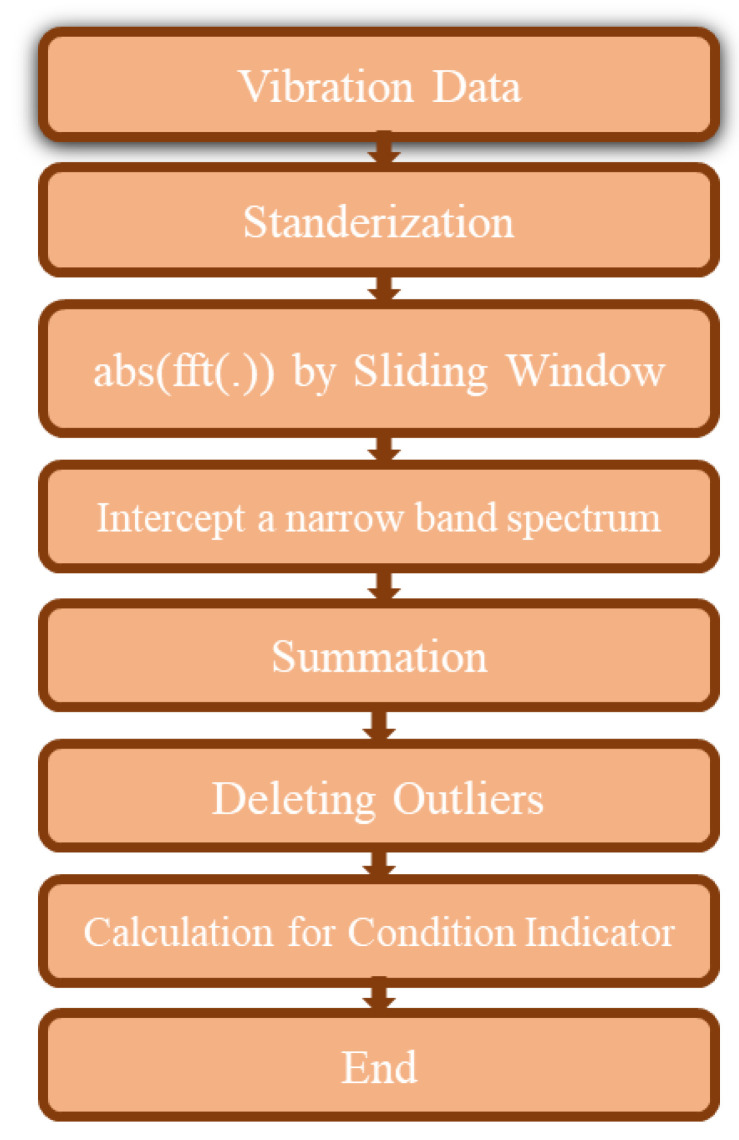
A flow chart showing the steps of the feature extraction procedure [5].

However, these datasets were not directly used for the health prognosis purpose. Since the slurry mixture, which was pumped using the considered slurry pump, had an indefinite amount of sand and tiny stones. As a result, vibration magnitudes obtained by mounted sensors also showed significant fluctuations. These fluctuations made the obtained vibration trends very difficult to observe as compared to linear and obvious trends associated with pure fluid pumps. Hence, the development of degradation trends of slurry pumps became a very challenging task. Actually, the random and uncertain fluctuations are the reason due to which traditional statistical methods cannot be applied for developing the performance degradation trends. In this regard, a specific procedure mainly consisting of the fast Fourier transform (FFT) technique for feature extraction was employed in this study. The procedure identified the salient feature(s) in raw vibration data for producing a progressive degradation trend for slurry pumps impellers. As shown in Figure 3, a series of eight steps as a feature extraction procedure was followed for extracting the fault indicator which can proceed with a progressive degrading pump and truly reflect the slurry pump’s health.

The procedures for extracting fault-related feature(s) from each slurry pump were identical. The details regarding the feature extraction process are as follows.

The vibration data **X**(*T*, *n*) was standardized as the first step using Equation (1) [5].

(1) $$X_{\mathrm{new}}\;\left(T,\;n\right)=\frac{X\;\left(T,\;n\right)mean\left(X\;\left(T,\;n\right)\right)}{rms\left(X_{\mathrm{new}}\;\left(T,\;n\right)\right)}$$

where,

*T* = pump measurement time in hours, (*T* = 1, 2, …, 1006 for first pump and 862 for second pump)

*n* = sample number index, (*n* = 1, 2, …, *N* where *N* = 51,200 for first pump and *N* = 60,000 for second pump)

mean (**X**(*T*, *n*)) = mean value of the elements in vector **X**(*T*, *n*), rms (**X**(*T*, *n*)) = returns the Root Mean Square, **X**_new_ (*T*, *n*) = standardized data.

The fast Fourier transform (FFT) technique converts the time-domain signals into frequency-domain signals and can thereby identify noticeable features in machines [5]. The characteristic frequencies of the slurry pumps were analyzed using FFT in this study. The averaged FFT amplitude value, **Y**(*T*, *f*), was obtained by utilizing the Fourier transform-based sliding window averaging technique using Equation (2) [5].

(2) $$Y\;\left(T,\;f\right)=\frac{1}{L}\;\overset{l+L}{\underset{T=l}{\sum }}│\overset{N-1}{\underset{n=0}{\sum }}X_{\mathrm{new}}\left(T,\;n\right)\exp(\frac{-i2\pi fn}{N})│$$

where, **Y**(*T*, *f*) is the averaged FFT amplitude value, *L* is window width, while *f* is frequency index. Afterward, within a narrow spectrum band, the averaged FFT amplitude values **Y**(*T*, *f*) were summed up and energy V(T) was calculated using Equation (3) [5]:

(3) $$V\left(T\right)=\overset{40}{\underset{f=19}{\sum }}Y\left(T,f\right)$$

where the energy V(T) was a substitute for the “rating frequency” of the vane passing frequency of the slurry pump under consideration. The overall frequency band was utilized for selecting the frequency band, which can be referred as the optimized frequency band. The frequency bands of all of the pump-measurement time were checked manually one by one. This activity was performed in order to make sure that all the operating situations had been taken into account. Ultimately, the sequential root mean square values, the *RMS* (*j*), were estimated by using Equation (4) [5]. Further details regarding this process can be seen in [5].

(4) RMS (j)=rms (V (1), V(2),…, V(j+q−1)

where,

*j* = file number index, (*j* = 1, 2, …, *K − q* + 1)

*q* = file numbers at the steady stage.

**Figure 4:**
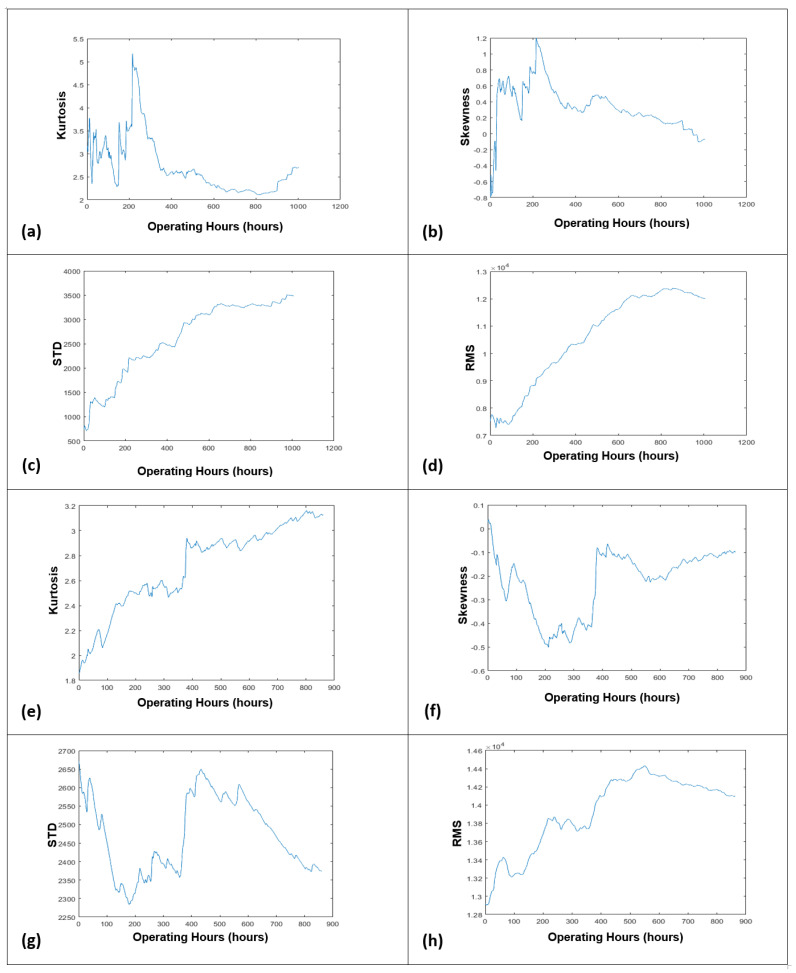
Degradation trends generated by kurtosis, skewness, St. deviation, and RMS using dataset 2014 (**a**–**d**), and dataset 2017 (**e**–**h**).

Based on the averaged FFT amplitude, the degradation trends that could reflect the progressing deterioration of the monitored slurry pump were developed by plotting the temporal increase of RMS features against the pump operation hours. Besides the RMS, the other commonly used time-domain features, like kurtosis, standard deviation, and skewness, were also utilized for reflecting the current health status of running slurry pumps, as shown in Figure 4.

However, the RMS feature best demonstrated the degrading situation of the deteriorating slurry pumps, among other features. It is a technical understanding that the pump’s impellers were new initially, so the degradation trend should be smooth at the start (or get degradation from the start, in the case if the pumped slurry is severely coarse). With the passage of time, when the impellers become older, they start degradation, so the degradation trend should be increased gradually and smoothly (or should be increased gradually with the deep crest and trough-like structures, in the case where the pumped slurry is very coarse). When the impellers get enough deterioration, they will stop offering resistance to slurry particles. In this situation, after reaching a particular height, the degradation trend should start to progress in a downward direction [9]. It can be observed in Figure 4. that only the degradation trends which have been developed using the RMS feature are satisfying the conditions of a meaningful degradation trend.

However, via following the mentioned procedure, two progressive degradation trends were generated, as shown in Figure 4d,h. After the development of degradation trends, the hybrid NAR-LSTM-BiLSTM model was developed and applied to learn the hidden characteristics of degradation trends for predicting the short-term and overall RULs of the slurry pumps. The design and working mechanism of the developed hybrid model are discussed below.

### 2.3. Design and the Working Mechanism of the Developed Model

**Figure 5:**
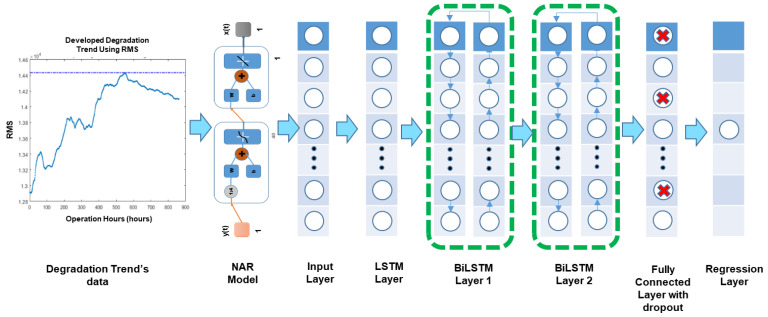
An illustrative diagram showing the overall architecture and data flow of the developed hybrid NAR-LSTM-BiLSTM model.

After generating the required degradation trends, the proposed hybrid NAR-LSTM-BiLSTM RUL prediction model was designed. At first NAR model was developed for which 70% of data was utilized for training purposes, 15% for validation, while 15% for testing purposes. The number of delays to calculate from the past information was taken as 4 while there were 40 neurons in the hidden layer. For the LSTM model, an adaptive moment estimation (Adam) training optimizer was selected since it is widely used due to its low memory requirement and high computational efficiency [10]. Maximum epochs were set at 300, and for preventing the overfitting issue during the training process, the dropout factor was set to 0.2. Figure 5 is shows the architecture of the developed model.

Since many researchers have favored Bayesian optimization (BO) for the optimization of hyperparameters like [11,12], therefore, the number of LSTM layers and their units, number of BiLSTM layers, and learning rate were selected using the Bayesian optimization (BO) method. It should be noted that the BO algorithm was individually run for 2014 and 2017 datasets. Consequently, different values of considered hyperparameters were selected for each case. For example, one BiLSTM layer, one LSTM layer, 248 LSTM units, and a learning rate of 0.041 were taken for the degradation trend developed from the 2014 dataset. Whereas two BiLSTM layers, one LSTM layer, 269 LSTM units, and a learning rate of 0.169 were chosen for the degradation trend developed from the 2017 dataset. The number of BiLSTM units was kept the same as the number of LSTM units.

It should be noticed that real-time online data is typically predicted for its RUL in “constructing a ladder” way for unsupervised data, i.e., (t)+(t)+(t+1), (t)+(t+1)+(t+2)…, and so on [13]. For instance, the NAR network takes a designated part of degradation trend as the training or input data, i.e., and after the first iteration, provides a one prediction point t+1. This prediction point, i.e., t+1, becomes the part of the training data in the form of (t)+(t+1) for the next iteration. Then after the second iteration, the NAR network again provides a prediction point, i.e., t+2. This process remains in progress until the desired number of prediction points are not achieved. Although, the simulation speed of the NAR network is very fast [14], but the problem attached with the NAR network is its issue of exploding gradient [15]. In this problem, the NAR model, which is providing its prediction points in a normal way, suddenly predicts a very large value than the previously acceptable prediction values/points. Since the NAR model perform its simulations at a very high speed [14], so it can be run many times until the prediction points do not come in the desired direction, i.e., towards the threshold point/line. On the other hand, the LSTM model also adopted the NAR model’s procedure for providing its prediction points. Though it simulates the iterations at a very slow speed, but it does not has an exploding gradient problem [16]. The problem that happened with the LSTM-BiLSTM model’s prediction points is their progression towards the downward direction rather than towards the threshold line/upward direction, just after the first/few iterations. Therefore, the developed hybrid model has compensated for the above-mentioned deficiencies of the individual NAR and LSTM-BiLSTM networks by complementing each of them.

## 3. Results and Discussion

**Figure 6:**
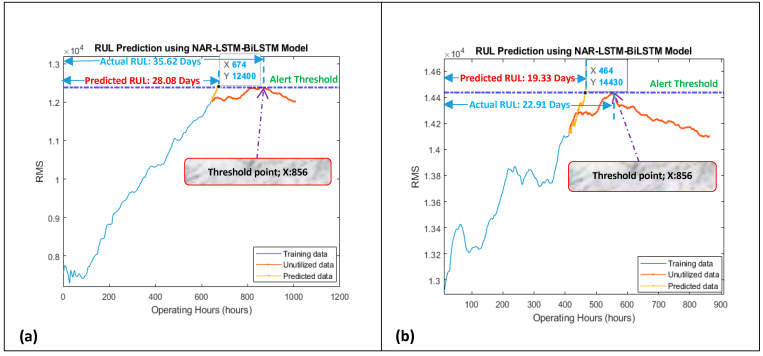
Prediction of overall RUL using 75% data of degradation trends for (**a**) dataset 2014 and (**b**) dataset 2017.

**Figure 7:**
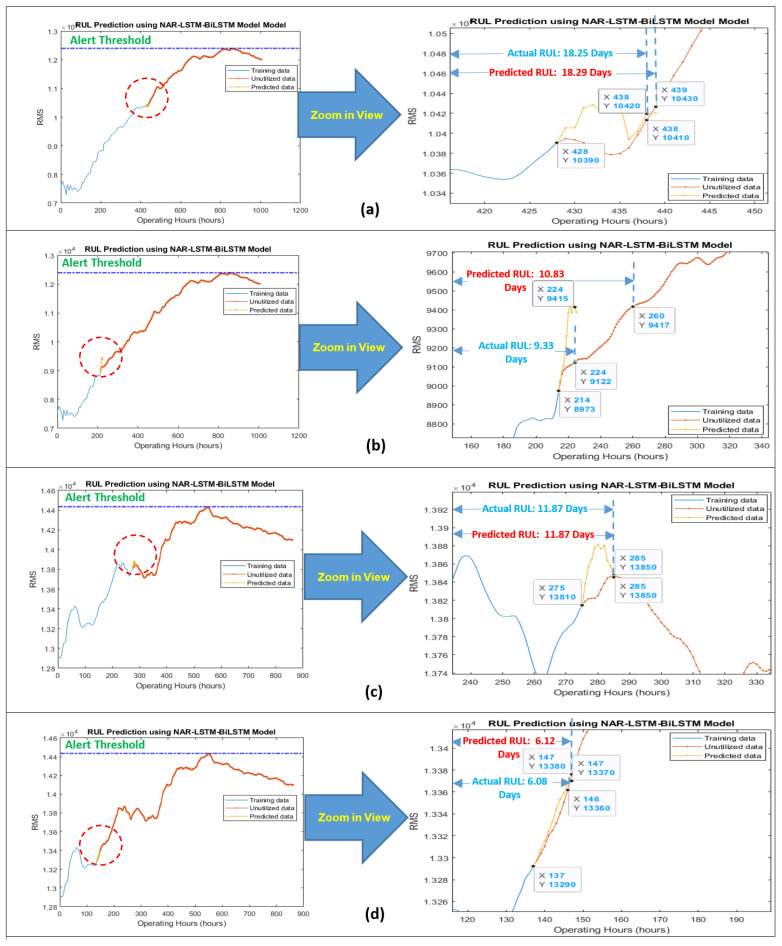
Prediction of short-term RUL for the next 10 h of the slurry pump using data sets 2014 for (**a**) 50% and (**b**) 25% data of degradation trends for training purpose, and dataset 2017 for (**c**) 50% and (**d**) 25% data of degradation trends for training purpose.

The data length of degradation trends from their start to the most heightened point (threshold point) was considered for modeling since the pump’s impellers typically started degradation after this point. The developed model was working in two steps. In the first step, the NAR network was utilized for obtaining a few prediction points in a correct direction, i.e., towards the threshold line. In the second step, the LSTM-BiLSTM model considered the NAR prediction results as the “path to be followed” for a few iterations (seven prediction points of the NAR model were picked up randomly in under-considered cases) for getting the correct direction for its prediction points, and then by taking the benefit of its own long-term memory, it produced outstanding regression prediction results for obtaining the overall RUL and short-term RULs, as shown in Figure 6 and Figure 7.

**Figure 8:**
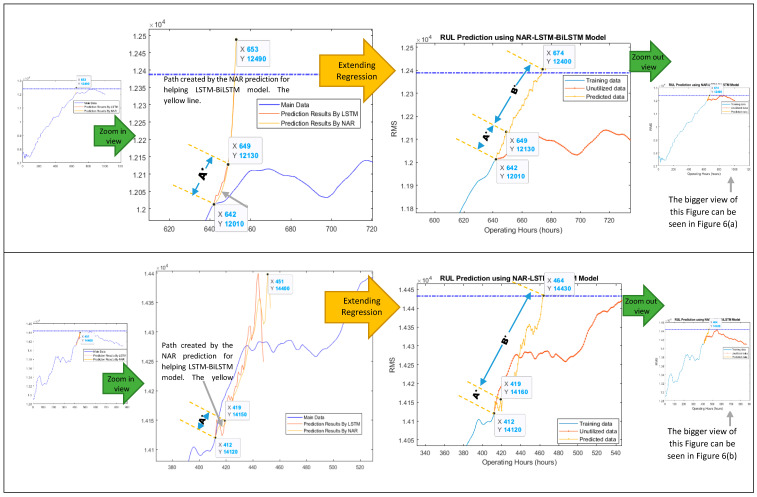
Explaining the RUL prediction philosophy by the hybrid NAR-LSTM-BiLSTM model for (**a**) datasets collected in 2014, and (**b**) datasets collected in 2017 for overall RUL prediction. A* = Initial LSTM-BiLSTM prediction points, which were obtained by following the path created by the NAR model. Actually, the path created by the NAR model is the prediction made by the NAR model. B* = Extending the A* for obtaining the overall RUL by utilizing the LSTM-BiLSTM model.

For obtaining the overall RUL, the LSTM-BiLSTM model’s prediction points follow the path created by the NAR model’s predictions and then iterate many times until the prediction points do not reach the threshold line. For obtaining the short-term RUL, the LSTM-BiLSTM model’s prediction points follow the path created by the NAR model’s predictions and then iterate for three more prediction points (since the short-term RUL was calculated for the next ten hours and every single iteration was producing one prediction point which was referred as the one hour). Figure 8 is showing the working mechanism of the developed approach for obtaining the overall RUL.

“A*”, is showing the prediction points which were obtained by the LSTM-BiLSTM model by following the NAR model’s prediction points. “B*”, is showing the LSTM-BiLSTM model’s prediction points which were obtained by further extending “A*”. While working for slurry pump having dataset of the year 2014, the developed model was predicting an overall RUL of 28.08 days against an actual RUL of 35.62 days, as shown in Figure 6a. These RULs were obtained by dividing x-coordinates values, i.e., 674 and 856, by 24. Hence it was found that via utilizing 75% data of degradation trend for training, the developed hybrid NAR-LSTM-BiLSTM model is generating its outcomes with an accuracy of 78.83%. In order to estimate the short-term RUL of a slurry pump having 2014 datasets, the developed hybrid NAR-LSTM model was fed with 50% and 25% data for its training. The model was run for ten iterations, or in other words, the RUL prediction results were drawn out for the next ten operating hours. In return, the developed hybrid NAR-LSTM model successfully revealed its results as a predicted RUL of 18.29 days and 10.83 days with an accuracy of 99.78% and 89.28%, respectively, as shown in Figure 7a,b. Similarly, when working for the slurry pump having datasets of the year 2017, the developed model was predicting an overall RUL of 19.33 days against actual RUL of 22.91 days, as shown in Figure 6b. Again, for predicting the short-term RUL of this slurry pump, the developed model was fed with 50 and 25% data of degradation trends for determining the RULs in the next ten operating hours., as shown in Figure 7c,d. It can be observed that the developed model’s average accuracy of the overall RUL prediction for both the slurry pumps was 81.60%, while the average accuracy for the short-term RUL prediction was 95.76%. Table 1 is showing the actual RULs, predicted RULs by the proposed model and Nonlinear Autoregressive Exogenous (NARX) model, and the accuracy of the obtained predicted RULs.

### 3.1. Validation of the Developed Methodology Using Other Channels

**Figure 9:**
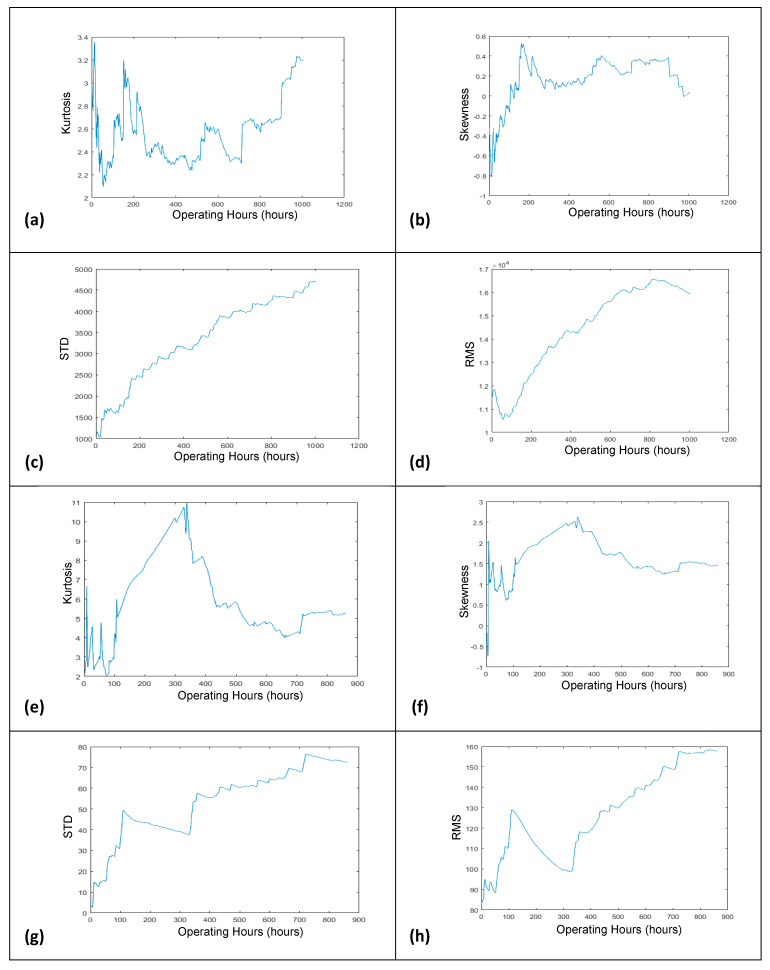
Degradation trends generated by kurtosis, skewness, St. deviation, and RMS using dataset 2014 (**a**–**d**), and dataset 2017 (**e**–**h**), for channel 4.

In order to verify the applicability of the developed strategy, it was also applied on the data which was obtained from sensor 4 of both the slurry pumps. As earlier, the obtained data was first cleaned, then was utilized for making the performance degradation trends via the proposed FFT method. It can be observed in Figure 9d that feature RMS has established a suitable degradation trend for dataset 2014, but for the dataset 2017, feature STD has created a more significant degradation trend, as shown in Figure 9g.

**Figure 10:**
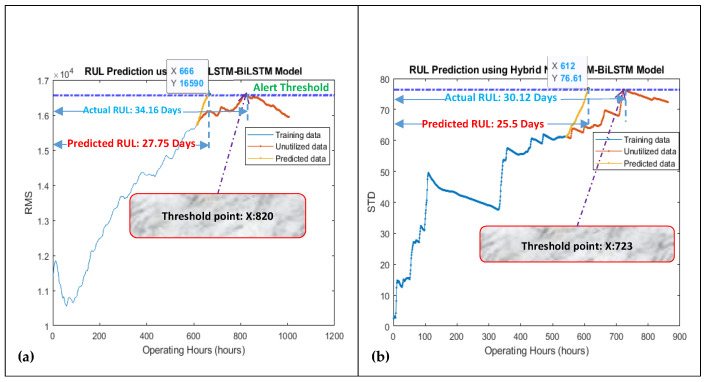
Prediction of overall RUL using 75% data of degradation trends for (**a**) dataset 2014, and (**b**) dataset 2017, for channel 4.

**Figure 11:**
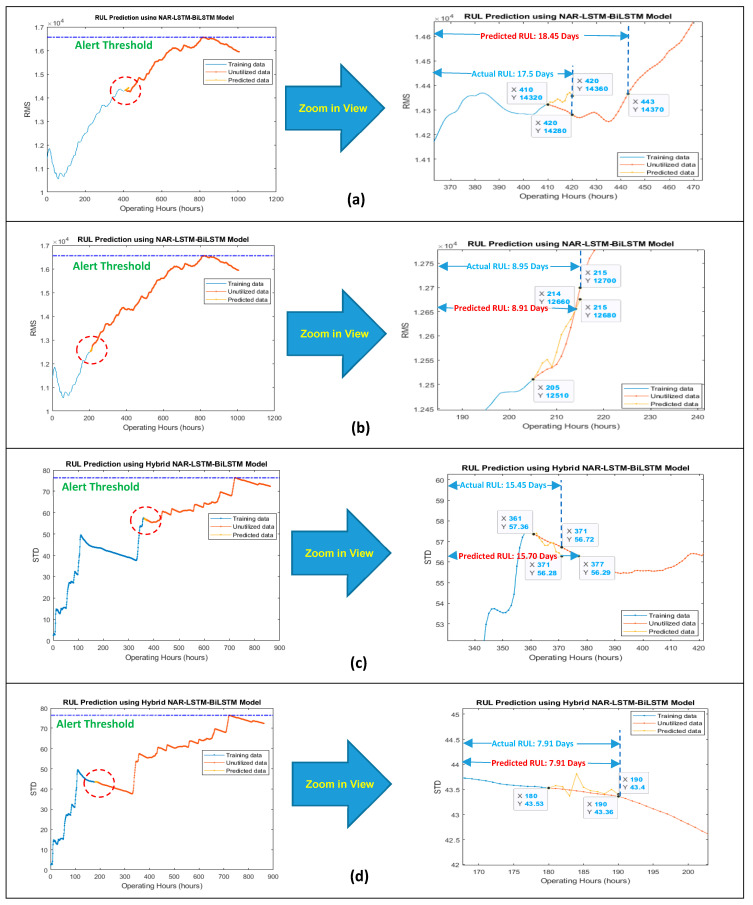
Prediction of short-term RUL for the next 10 h of the slurry pump using data sets 2014 for (**a**) 50% and (**b**) 25% data of degradation trends for training purpose, and dataset 2017 for (**c**) 50% and (**d**) 25% data of degradation trends for training purpose.

The dynamics of an expressive performance degradation trend have already been discussed in Section 2.2 of the paper. Therefore, unlike before, the degradation trend built-up by the feature STD was selected for dataset 2017, for further simulation. Figure 10, Figure 11 and Table 2 show the overall and short-term predicted RULs obtained by the developed hybrid NAR-LSTM-BiLSTM model. It was noticed that the developed model was predicting its overall RUL with an average accuracy of 82.94%, while short-term RUL with an average accuracy of 98.19%.

### 3.2. Validation of the Developed Methodology Using C-MAPSS Dataset

In order to verify the working mechanism of the developed methodology, it was also applied on the publically available NASA Commercial Modular Aero Propulsion System Simulation (C-MAPSS) dataset. It was employed for estimating the RUL of two individual turbofan jet engines in a novel way, i.e., without using their available run to failure datasets as per the concept explained in Section 1. The C-MAPSS dataset consists of four sub-datasets named as FD001, FD002, FD003, and FD004 with the different or same number of train/test trajectories and operation/fault modes. Each sub-dataset comprises multiple multivariate time series. There are 26 columns which correspond to, (i) engine number, (ii) time (cycles), (iii) operation specifications 1, (iv) operation specifications 2, (v) operation specifications 3, (vi) to (xxvi) Data obtained by sensor 1, to data obtained by sensor 21.

Each row is a snapshot of data taken for a single operational cycle, while each time series is from a different engine. It means that the data can be considered from a fleet of identical engines. Each engine has started with different manufacturing variations and initial wear that is not known to the user. However, this variation and wear is normal, and it is not considered as the fault condition. At the start of each time series, the engines are operating normally, and then they get a fault at some point during their operations. In the training trajectories, there is data where the fault grows in magnitude until it makes the system fail, i.e., run to failure data. In the test trajectories, there is the time-series data that ends sometime prior to the system failure, i.e., run to prior failure data. The dataset has also provided the true values of remaining operational cycles, i.e., actual RUL of the engines. However, the time-series data for the actual remaining operational cycles is not given. Traditionally, researchers utilized the run to failure data for the development of their RUL prediction models and then test their developed model’s results with the provided run to prior failure dataset. In the world of research, this methodology is acceptable, and a lot of research papers can be found on this topic from the literature. However, as discussed earlier, in the real world, it is impossible to have a huge amount of run to failure data from a fleet of identical rotary machines (just like in the case of the C-MAPSS dataset). In industry, operating machines are never run until their failure since they are typically provided with the TBM. Keeping the situation in view, the RUL of two individual engines of the CMAPSS dataset by only utilizing their run to prior failure data has been predicted in this study.

**Figure 12:**
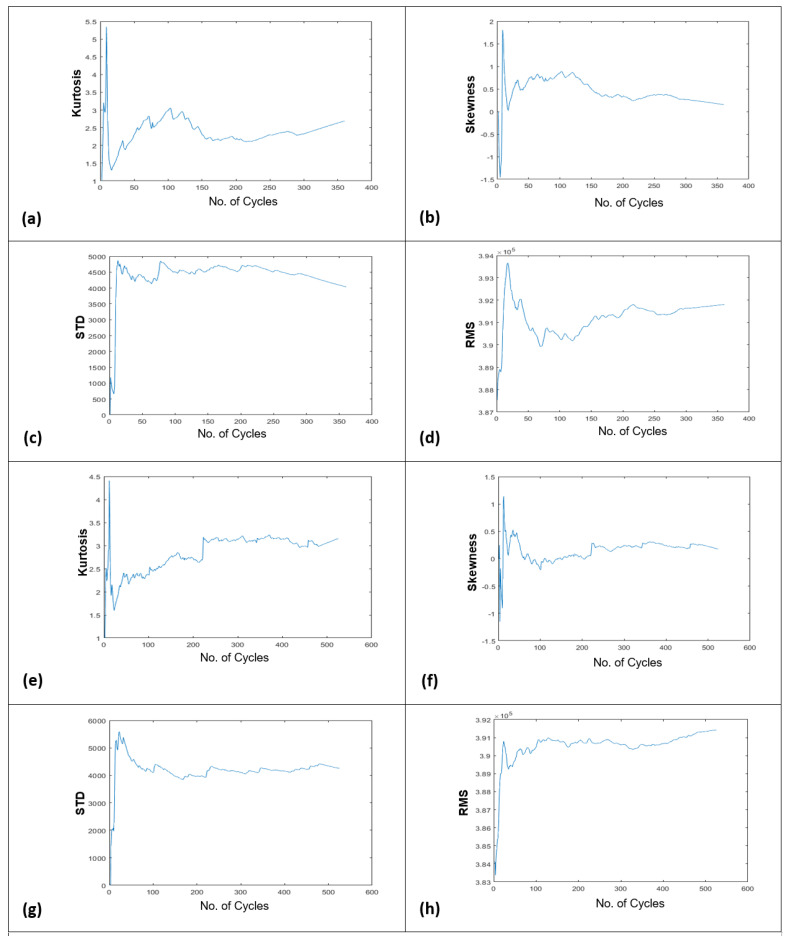
Degradation trends generated by using kurtosis, skewness, St. deviation, and RMS using sub-dataset FD002 for engine 140 (**a**–**d**), and sub-dataset FD004 for engine 25 (**e**–**h**).

Engine No. 140 from sub-dataset FD002 and engine No. 25 from sub-dataset FD004, were selected for predicting their RULs. Engine 140 has 306 rows of time series from 26 sensors in the package of the run to prior failure data. Since only a constant value of final RUL, i.e., 55 cycles, is given in the dataset, and no time-series data has been provided for these 55 cycles. Therefore, the 306 rows of sensor data were extrapolated to a further 55 rows, which gave a sum of 361 rows. It implies that the total life of particularly this engine is 361 cycles. Via applying the proposed FFT method on these 361 rows (which are actually 361 operational cycles of this engine) of data, four different performance degradation trends using the time-based indicators, i.e., kurtosis, skewness, standard deviation, and root mean square were developed, as shown in Figure 12a–d.

**Figure 13:**
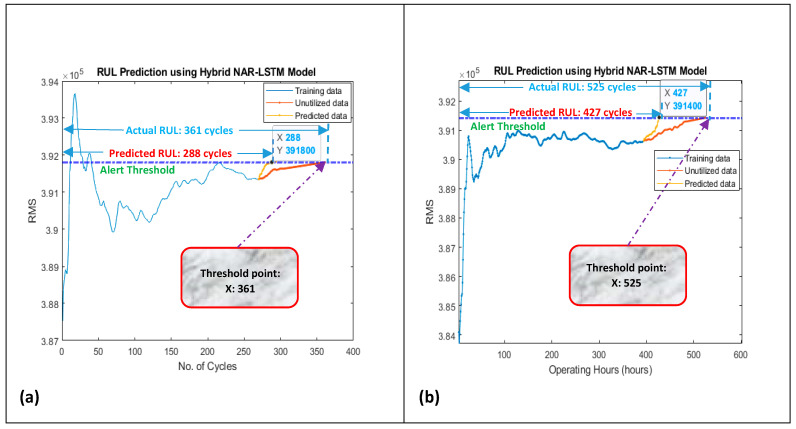
Prediction of overall RUL using 75% data of degradation trends of (**a**) engine No. 140 from FD002 and (**b**) engine No. 25 from FD004, for training.

Similarly, engine No. 25 has 486 rows of time series data (i.e., 486 number of cycles before failure), while its provided actual RUL value is 39 cycles in the dataset. Same like earlier, the given time series data, i.e., 486 rows of data, were extrapolated to 39 more rows, which gave a sum of 525 rows. It implies that the total life of particularly this engine is 525 cycles. By applying the proposed FFT method on these 525 operational cycles, four degradation trends were also developed for this engine, as shown in Figure 12e,f. Degradation trends that were obtained by using the RMS indicator were selected for predicting the overall and short-term RULs. The threshold line was drawn with reference to the last value of the degradation trend since it was the given threshold point in the dataset. Later on, the developed NAR-LSTM-BiLSTM model was utilized for predicting the overall and short-term RULs for both the engines in the same way as for the slurry pumps. In the case of engine 140, an overall RUL of 288 cycles was predicted against its total real life of 361 cycles, as shown in Figure 13a.

**Figure 14:**
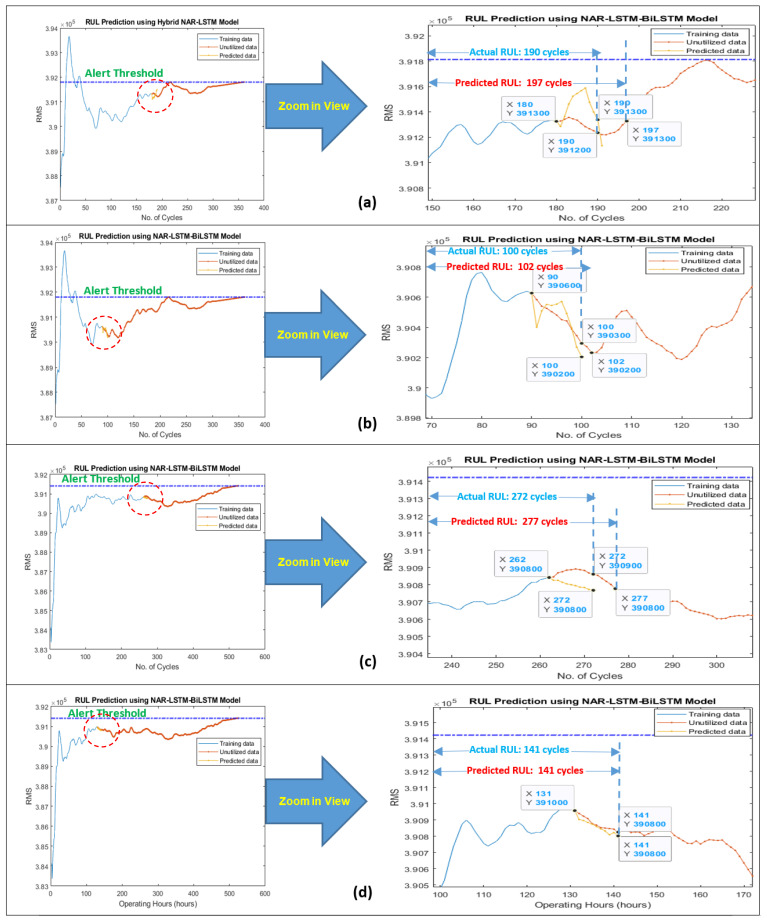
Prediction of short-term RUL for the next 10 h of turbofan jet engines using FD002 for (**a**) 50% and (**b**) 25% data of engine No. 140 degradation trends and FD004 for (**c**) 50% and (**d**) 25% data of engine No. 25 degradation trends.

Similarly, in the case of engine No. 25, an overall RUL of 427 cycles was predicted while its total actual life was 525 cycles, as shown in Figure 13b. Collectively the accuracy for both the overall RUL predictions was found to be 80.55%. The developed model was again utilized for predicting the short-term RUL of the under-considered engines. RUL for the next 10 cycles was predicted using 50 and 25% data of their degradation trends, and the results were obtained with an averaged accuracy of 98.11% for short-term RULs, as shown in Figure 14.

Table 3 is showing the actual RULs, predicted RULs by the proposed model, NARX, and the LSTM model, and the accuracy of the obtained predicted RULs for the engine’s operational cycles.

## 4. Conclusions

Recently established RUL prediction methods are developed on the basis of a perfect database which is typically comprised of a huge “run to failure” and “run to prior failure data”. However, in the real-world, run to failure data of rotary machines is not available due to the regular practice of the TBM on machines. Keeping the situation in view, a unique strategy for RUL prediction using only run to prior failure data of in operation slurry pumps was developed in this research. In order to cross verify the developed methodology, it was also applied on (i) other channels of the slurry pumps, and (ii) two individual engines of publically available C-MAPSS dataset. The results obtained by the developed model were quite satisfactory. In this study,

(I) Initially, the data was analyzed and, an FFT-based method that can produce the performance degradation trends of the monitored slurry pumps was presented. Then, the architecture of a novel hybrid NAR-LSTM model for predicting the RUL of the deteriorating slurry pumps was designed. Since the NAR model has an exploding gradient problem while the LSTM network is slow, along with the problem of wrong progression direction of prediction points, the proposed model was developed, which took advantage of (i) the fast simulation speed of the NAR model for getting a correct direction, i.e., towards the threshold line and (ii) long term memory power of the LSTM-BiLSTM network for avoiding exploding gradient problem. Initial prediction points of the NAR model were utilized as the “path to be followed” by the LSTM-BiLSTM model for providing its own prediction points, which were ultimately meeting the threshold line. The RUL prediction results yielded by the developed model also dominated over the results generated by the other online RUL prediction techniques.
(II) Existing RUL prediction models available in the literature are only working for the slurry pumps, which have very smooth degradation trends. The developed model is a robust model which is predicting the RUL of the slurry pumps satisfactorily either the degradation trend is progressing in a smooth way, or it has many deep crest and trough-like structures. Similarly, the existing methods are only predicting the overall RUL, while the developed model is also estimating the short-term RULs along with the overall RUL of the slurry pumps with acceptable accuracy.
(III) This research has also reflected a novel aspect and usage of the C-MAPSS dataset for predicting the online RULs of the individual turbofan jet engines via utilizing only their run to prior failure data. It is anticipated that if more amount of data like 80% or 90% of degradation trend’s data will be utilized for training of the developed model, the results will be more accurate. However, at the same time, it will also increase the risk of the breakdown of the considered slurry pump or engine.

The authors are making efforts for further improvement of RUL prediction results. The developed model can also be modified for predicting the RULs of other types of rotary machines that show substantial fluctuations in measured signals, like vibration, forces, pressure, etc.

## Figures and Tables

**Table 1:** The actual RULs, predicted RULs by the NARX and the developed model, and the accuracy of the obtained predicted RULs for slurry pumps.

2014 Datasets				
	Training Data (%)	RUL Predicted (Days)	Actual RUL (Days)	Accuracy of Predicted RUL (%)
Overall RUL				
Proposed Method	75	28.08	35.62	78.83
NARX	75	27.10	35.62	76.08
Short-term RUL				
Proposed Method	50	18.29	18.25	99.78
NARX	50	17.23	18.25	94.41
Proposed Method	25	10.83	9.33	83.92
NARX	25	11.01	9.33	81.99
2017 Datasets				
Overall RUL				
Proposed Method	75	19.33	22.91	84.37
NARX	75	Not applicable	22.91	-
Short-term RUL				
Proposed Method	50	11.87	11.87	100
NARX	50	11.34	11.87	95.53
Proposed Method	25	6.12	6.08	99.34
NARX	25	5.64	6.08	92.76

**Table 2:** The actual RULs, predicted RULs by the NARX and the developed model, and the accuracy of the obtained predicted RULs for slurry pumps.

2014 Datasets				
	Training Data (%)	RUL Predicted (Days)	Actual RUL (Days)	Accuracy of Predicted RUL (%)
Overall RUL				
Proposed Method	75	27.75	34.16	81.23
NARX	75	26.60	34.16	77.86
Short-term RUL				
Proposed Method	50	17.5	18.45	94.85
NARX	50	17.40	18.45	94.25
Proposed Method	25	8.91	8.95	99.55
NARX	25	8.68	8.95	96.98
2017 Datasets				
Overall RUL				
Proposed Method	75	25.5	30.12	84.66
NARX	75	Not applicable	30.12	-
Short-term RUL				
Proposed Method	50	15.70	15.45	98.38
NARX	50	15.76	15.45	97.99
Proposed Method	25	7.91	7.91	100
NARX	25	7.90	7.91	99.87

**Table 3:** The actual RULs, predicted RULs by the LSTM, NARX and the developed model, and the accuracy of the obtained predicted RULs for turbofan jet engines.

Engine No. 140 from Sub-Dataset FD002				
	Training Data (%)	RUL Predicted (Cycles)	Actual RUL (Cycles)	Accuracy of Predicted RUL (%)
Overall RUL				
Proposed Method	75	288	361	79.77
NARX	75	274	361	75.90
LSTM	75	Not applicable	361	-
Short-term RUL				
Proposed Method	50	197	190	96.31
NARX	50	200	190	94.73
LSTM	50	196	190	96.93
Proposed Method	25	102	100	98.00
NARX	25	104	100	96.00
LSTM	25	103	100	97.08
Engine No. 25 from sub-dataset FD004				
Overall RUL				
Proposed Method	75	427	525	81.33
NARX	75	Not applicable	525	-
LSTM	75	Not applicable	525	-
Short-term RUL				
Proposed Method	50	277	272	98.16
NARX	50	280	272	97.05
LSTM	50	278	272	97.84
Proposed Method	25	141	141	100
NARX	25	146	141	96.45
LSTM	25	144	141	97.91

## Data Availability

The slurry pumps datasets which have been used in this study are not available for public use. The C-MAPSS datasets presented in this study are openly available in NASA Ames Prognostics Data Repository at http://ti.arc.nasa.gov/project/prognostic-data-repository accessed on 1 December 2021.

## Supplementary Materials

The following are available online at https://www.mdpi.com/article/10.3390/s21248420/s1, Section S1. Background; Section S2. Current Research Work with NAR and LSTM Models for Solving the Engineering Problems.
