# Supplementary material for: Development of a Novel Methodology for Remaining Useful Life Prediction of Industrial Slurry Pumps in the Absence of Run to Failure Data
Source: Sensors (Basel). 2021 Dec 16;21(24):8420. doi: 10.3390/s21248420 (PMC8715750; doi:10.3390/s21248420)
Supplement: Supplementary file 1 [file sensors-21-08420-s001.zip › sensors-1456287-supplementary.pdf]

Supplementary Work

S.1. Background

S.1.1. Nonlinear Autoregressive (NAR) Neural Network

The Nonlinear Autoregressive (NAR) model is used for interpolation, regression, and discrete-time series prediction. NAR neural network is a discrete model comprising of an input layer, input delay, hidden layer, an output layer, and output delay [1]. The network weights and neurons are optimized during the training phase using the Levenberg-Marquardt algorithm. Adjustment of hidden layers' errors is performed by back-propagating the error of the outer layer to further backward layers, i.e., input layers. There are two steps in the algorithm. In the first step, the input is handled for forward propagation of information, where it is processed one by one at each unit from the input layer to the hidden layer. In the second stage, the reverse flow of the information is being made for minimizing the error to its least value [1]. The mathematical formulation followed by the NAR network is given in equation (S.1) [2]. Further details can be seen in [1] and [2].

$$x(t) = f\big(y(t-1) + y(t-2) + \cdots + y(t-d)\big) + \varepsilon(\mathrm{t}) \quad \text{(S1)}$$

$where$
$f = the\ unknown\ nonlinear\ function\ that\ can\ be$
$approximated\ by\ the\ feedforward\ neural\ networks$
$x(t) = the\ predicted\ value\ of\ the\ data\ series\ of$
$y\ at\ a\ discrete\ time\ step\ t$
$d\ =\ past\ values\ of\ the\ series$
$\varepsilon(\mathrm{t}) = the\ approximation\ error\ of\ the\ series\ y\ at\ timet$

S.1.2. Long short-term memory networks and Bidirectional LSTMs

Long Short-Term Memory (LSTM) network is a type of Recurrent Neural Networks (RNN) as it comprises a recursive property similar to that of RNN. This network has a unique ability of memory and forgetting mode, i.e., adapted by the timing characteristics for performing the learning tasks. Within that, LSTM also solves the problem of vanishing and exploding gradient using the backpropagation through time (BPTT) training process, which was not solved by the standard RNN. This ability makes the most use of hidden information and the time-dependent relationship in sequence data. Unlike conventional RNN, the hidden layer of LSTM is not a common neural unit, but it is unit that has a unique memory system, as shown in Figure 1. A number of operations are performed inside of a single LSTM unit for generating an output value. There are three gates named as input, forget and output gate which helps in producing the output by neglecting the unnecessary data and only keep the information which is important for minimizing the error of prediction [3]. Equations (S.2),(S.3), ans (S.4) are related with these gates [4]. These gates are typically described by the sigmoid functions [5]. Figure S1. is showing the basic architecture of NAR and LSTM networks with the

representation of their mathematical computations [5]. Further details can be seen in [3], and [4].

$$f_t = \sigma \ (x_t \times U_t \times h_{t-1} \times W_f) \tag{S2}$$

$$I_t = \sigma \ (x_t \times U_i \times h_{t-1} \times W_t) \tag{S3}$$

$$o_t = \sigma \ (x_t \times U_o \times h_{t-1} \times W_o) \tag{S4}$$

where
h_t = *the hidden state of the current time stamp*
h_{t-1} = *the hidden state of the previous time stamp*
C_t = *the cell state of the current time stamp*
C_{t-1} = *the cell state of the previous time stamp*
x_t = *input to the current timestamp*
U_t, U_i, U_o = *weight associated with the input*
W_f, W_t, W_o = *the weight matrix associated with hidden state*

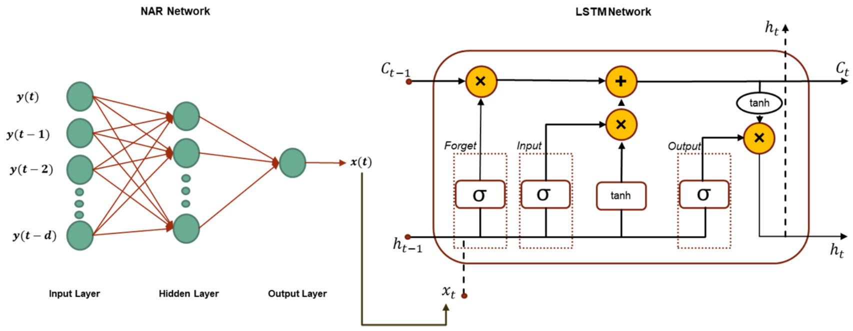

Figure S1. The basic architecture of NAR and LSTM networks with the representation of their mathematical computations [5].

Bidirectional LSTM networks have an inverse learning mechanism while comparing with RNN and LSTM. For the optimization of the output results, BiLSTM also considers future information along with historical knowledge of data. In a basic BiLSTM network, there are two independent layers of LSTMs; one of them is used for forward training while the other for reverse training of the network. The final result of BiLSTM is actually a combined output result of the two forward and backward LSTM network layers [6].

S.2. Current Research Work with NAR and LSTM Models for Solving the Engineering Problems

Ibrahim et al. [7] utilized NAR and Discrete Wavelet Transform (DWT) for increasing the life of the battery. He developed a new energy management strategy by utilizing real data of a military hybrid vehicle. Chun Suet et al. [8] investigated the batteries historical operation data and then built four different types of health indicators for them. He utilized a generalized regression neural network (GRNN), and NAR to predict the batteries online RUL. Zhang et al. [9] worked for the RUL prediction of lithium-ion batteries. He employed LSTM for learning the capacity degradation trajectories of batteries. He used the resilient mean square back-propagation method for adaptively optimizing his

LSTM model. Ali et al. [10] developed a CNN-LSTM Hybrid Deep Neural Network Model (HDNN), which estimated the RUL using two deep learning models concurrently for the very first time. He tested his HDNN model on the NASA commercial modular aero-propulsion system simulation (C-MAPSS) dataset, and his results outperformed other traditional techniques. Zhanga et al. [11] developed a new method using LSTM. For explaining the system health condition, he translated the raw sensor data to an interpretable health index. He also tracked the historical degradation of the system for predicting its future health condition. He used NASA's C-MAPSS dataset for verification of his method. His developed method simply outperformed other traditional methods. Wang et al. [12] developed a new approach of RUL prediction via utilizing the Bidirectional Long Short-Term Memory (BiLSTM) network. He experimented with his model with the CMAPSS dataset and proved that his model is better than other traditional approaches of RUL estimation in terms of accuracy. Trappey, A. et al. [13] performed real time monitoring of main parameters of power transformers using data mining for their fault predictions. She utilized a combination of Principal component analysis (PCA) and a back-propagation artificial neural network (BP-ANN) for the prediction model. Wentao et al. [14] utilized support vector data normalized correlation coefficient for dividing the whole life of bearings into fast and normal degradation state. Then he used CNN for transforming health state labels and raw vibration signals. Ultimately he utilized LSTM for RUL prediction and experimented on bearing data sets of IEEE PHM Challenge 2012. Peng Shi et al. [15] developed a hybrid LSTM-CNN model for material fatigue RUL prediction. He tested his model on 1193 groups of carbon steel fatigue data set and found promising results. Hai Li et al. [16] developed a deep bidirectional Long Short Term Memory (DBiLSTM) network model for predicting the RUL of the milling machine tool with limited data. He verified the effectiveness of his formulated DBiLSTM by conducting experiments on several workpieces of stainless steel using the milling machine tool. Fu-Kwun et al. [17] developed a bi-directional LSTM model with an attention mechanism (BILSTM-AT) for predicting the voltage degradation of Proton exchange membrane fuel cells (PEMFCs). He used the Random forest regression model for extracting the important variables as the inputs for his model and then utilized the sliding window method for predicting the RUL of PEMFCs.

Thus, the literature review revealed that NAR, LSTM, and BiLSTM are such strong algorithm that have been used individually or with some other hybrid combinations for many engineering applications like batteries [7–9], aircraft turbofan jet engines [10–12], bearings [14], carbon steel fatigue [15], milling machine tool [16], proton exchange membrane fuel cell [17], etc. But as per the author's best knowledge, no one has used them as a combination via making a hybrid model for the RUL prediction. Keeping in view all the above-given literature, the NAR-LSTM-BiLSTM modeling was performed for the RUL prediction of available slurry pumps.

References

[1] A. H. Bukhari, M. Sulaiman, M. A. Z. Raja, S. Islam, M. Shoaib, and P. Kumam, "Design of a hybrid NAR-RBFs neural network for nonlinear dusty plasma system," *Alexandria Eng. J.*, 2020, doi: 10.1016/j.aej.2020.04.051.

[2] R. Taherdangkoo, A. Tatomir, M. Taherdangkoo, P. Qiu, and M. Sauter, "Nonlinear autoregressive neural networks to predict hydraulic fracturing fluid leakage into shallow groundwater," *Water (Switzerland)*, vol. 12, no. 3, 2020, doi: 10.3390/w12030841.

[3] H. Huu Son, "Toward a proposed framework for mood recognition using LSTM Recurrent Neuron Network," in *Procedia Computer Science*, 2017, doi: 10.1016/j.procs.2017.05.378.

[4] Shipra Saxena, "Introduction to Long Short Term Memory (LSTM)." [Online]. Available: https://www.analyticsvidhya.com/blog/2021/03/introduction-to-long-short-term-memory-lstm/. [Accessed: 22-Nov-2021].

[5] G. Rojas-Dueñas, J.-R. Riba, M. Moreno-Eguilaz, and M. G. Xibilia, "CNN-LSTM-Based Prognostics of Bidirectional Converters for Electric Vehicles' Machine," *Sensors 2021, Vol. 21, Page 7079*, vol. 21, no. 21, p. 7079, Oct. 2021, doi: 10.3390/S21217079.

[6] Z. Wang, J. Qu, X. Fang, H. Li, T. Zhong, and H. Ren, "Prediction of early stabilization time of electrolytic capacitor based on ARIMA-Bi_LSTM hybrid model," *Neurocomputing*, vol. 403, pp. 63–79, 2020, doi: 10.1016/j.neucom.2020.03.054.

[7] M. Ibrahim, S. Jemei, G. Wimmer, and D. Hissel, "Nonlinear autoregressive neural network in an energy management strategy for battery/ultra-capacitor hybrid electrical vehicles," *Electr. Power Syst. Res.*, vol. 136, pp. 262–269, 2016, doi: 10.1016/j.epsr.2016.03.005.

[8] C. Su, H. Chen, and Z. Wen, "Prediction of remaining useful life for lithium-ion battery with multiple health indicators," *Eksploat. i Niezawodn.*, vol. 23, no. 1, pp. 176–183, 2021, doi: 10.17531/EIN.2021.1.18.

[9] Y. Zhang, R. Xiong, H. He, and Z. Liu, "A LSTM-RNN method for the lithuim-ion battery remaining useful life prediction," in *2017 Prognostics and System Health Management Conference, PHM-Harbin 2017 - Proceedings*, 2017, doi: 10.1109/PHM.2017.8079316.

[10] A. Al-Dulaimi, S. Zabihi, A. Asif, and A. Mohammadi, "A multimodal and hybrid deep neural network model for Remaining Useful Life estimation," *Comput. Ind.*, 2019, doi: 10.1016/j.compind.2019.02.004.

[11] J. Zhang, P. Wang, R. Yan, and R. X. Gao, "Long short-term memory for machine remaining life prediction," *J. Manuf. Syst.*, 2018, doi: 10.1016/j.jmsy.2018.05.011.

[12] J. Wang, G. Wen, S. Yang, and Y. Liu, "Remaining Useful Life Estimation in Prognostics Using Deep Bidirectional LSTM Neural Network," in *Proceedings - 2018 Prognostics and System Health Management Conference, PHM-Chongqing 2018*, 2019, doi: 10.1109/PHM-Chongqing.2018.00184.

[13] A. J. C. Trappey, C. V. Trappey, L. Ma, and J. C. M. Chang, "Intelligent engineering asset management system for power transformer maintenance decision supports under various operating conditions," *Comput. Ind. Eng.*, vol. 84, pp. 3–11, 2015, doi: 10.1016/j.cie.2014.12.033.

[14] W. Mao, J. He, J. Tang, and Y. Li, "Predicting remaining useful life of rolling bearings based on deep feature representation and long short-term memory neural network," *Adv. Mech. Eng.*, 2018, doi: 10.1177/1687814018817184.

[15] P. Shi, L. Hong, and D. He, "Using Long Short Term Memory Based Approaches for Carbon Steel Fatigue Remaining Useful Life Prediction," in *Proceedings - 2018 Prognostics and System Health Management Conference, PHM-Chongqing 2018*, 2019, doi: 10.1109/PHM-Chongqing.2018.00187.

[16] H. Li, W. Wang, Z. Li, L. Dong, and Q. Li, "A novel approach for predicting tool remaining useful life using limited data," *Mech. Syst. Signal Process.*, vol. 143, p. 106832, 2020, doi:

10.1016/j.ymssp.2020.106832.

[17] F. K. Wang, T. Mamo, and X. Bin Cheng, “Bi-directional long short-term memory recurrent neural network with attention for stack voltage degradation from proton exchange membrane fuel cells,” *J. Power Sources*, vol. 461, no. April, p. 228170, 2020, doi: 10.1016/j.jpowsour.2020.228170.
